# Child Sexual Abuse—Challenging Conditions for Adult Oral Health: A Qualitative Study

**DOI:** 10.1177/23800844211053775

**Published:** 2021-10-27

**Authors:** E. Wolf, S. Månsson, L. Wallin, G. Priebe

**Affiliations:** 1 Department of Endodontics, Faculty of Odontology, Malmö University, Malmö, Sweden; 2 Department of Psychology, Lund University, Lund, Sweden

**Keywords:** access to care, anxiety, dental fear, dental public health, dental care, child molestation

## Abstract

The aim was to analyze perceptions of oral health in adults who have been exposed to child sexual abuse. Eleven participants (10 women), 19 to 56 y of age, who had experienced sexual abuse as children were purposively selected and interviewed in-depth. The participants were encouraged to describe how they perceived the effect of the sexual abuse on their oral health as adults. The interviews were recorded digitally and transcribed verbatim. The collected material was analyzed according to qualitative content analysis. The theme “challenging conditions for maintaining oral health” was identified, comprising 2 categories: first, “the emotional significance,” with the subcategories 1) emotional barriers and 2) powerful relief, and second, “the obstacles to oral health,” with the subcategories 1) daily self-care with complications and 2) dental appointments with difficulties. The findings indicate that the experience of sexual abuse during childhood can have a negative impact on oral care in adulthood. The informants stated that oral health was of utmost importance but also associated with strong emotions. There were obstacles to maintenance of oral health that were difficult to surmount.

Knowledge Transfer Statement:The study provides access to the attitudes of survivors of child sexual abuse regarding oral health and the needs and obstacles that they experience. This is important knowledge for dental professionals to optimize dental care.

## Introduction

On January 1, 2020, the Convention on the Rights of the Child became law in Sweden. Article 19 states the right of children to be protected from all forms of violence, abuse, and neglect (UNICEF 1990). In its comment on article 19, the Committee on the Rights of the Child describes sexual abuse as

a) the inducement or coercion of a child to engage in any unlawful or psychologically harmful sexual activity, b) the use of children in commercial sexual exploitation, c) the use of children in audio or visual images of child sexual abuse and d) child prostitution, sexual slavery, sexual exploitation in travel and tourism, trafficking (within and between countries) and sale of children for sexual purposes and forced marriage. Many children experience sexual victimization which is not accompanied by physical force or restraint but which is nonetheless psychologically intrusive, exploitive and traumatic. (UNICEF 1990)

Sexual violence might be regarded as one of the most profound violations of human rights (Jeong and Cha 2019) and is considered a public health problem (Krug et al. 2002). Child sexual abuse (CSA) can have short- and long-term physical and mental sequelae (Paras et al. 2009; Hailes et al. 2019), incurring considerable expense for the individual and society (National Board of Health and Welfare 2006).

An association between exposure to CSA and the development of dental fear has been shown (Willumsen 2001; Leeners et al. 2007). It is common for patients with dental fear to seek treatment only in case of emergency (Hakeberg et al. 2000), which easily leads to a vicious cycle: the dental status deteriorates; the need for treatment increases; and the treatment required is more difficult and complicated (Armfield et al. 2007).

The World Dental Federation states that “oral health is multifaceted and includes the ability to speak, smile, smell, taste, touch, chew, swallow, and convey a range of emotions through facial expressions with confidence and without pain, discomfort, and disease of the craniofacial complex” (FDI 2016). The perception of oral health by adults who have experienced CSA is poorly documented.

When a phenomenon is studied in its own context and when an understanding of thoughts, emotions, attitudes, and perceptions is desirable, a qualitative research approach is considered suitable (Stewart et al. 2008).

The aim of the present study was to analyze perceptions of oral health in adults who have been exposed to CSA.

## Material and Method

### Context

The Swedish Public Dental Health Service is financed by a combination of taxes and patient fees (Koch 2014). According to the legislation (“SFS 1985:125” 1985), the aim of Swedish dental care is to ensure good oral health for the population. Dental care shall be knowledge based and appropriate, safe, patient focused, and efficient. Access to care shall be equal and provided within a reasonable time. Patients are free to choose their care provider, either the Public Dental Health Service or a private practitioner. In adult dental care, fees are not fixed, and the patients bear a significant part of the cost. Until recently, dental care for children and young people has been free of charge until the age of 19 y. This has now been extended until the year they turn 23. Approximately 40% of the total Swedish population is treated by the Public Dental Health Service, including the majority of children (Tandvårds-och läkemedelsförmånsverket 2020).

### Qualitative Content Analysis

This method comprises an inductive approach and is a systematic scrutiny of text resulting in a categorization of patterns: the manifest content (the contents’ descriptive level). The manifest content is reflected on and a theme is formulated illustrating this interpretative level: the latent content. The method enables the researcher to explore topics chosen by the informant and not previously considered or driven by a researcher’s preconceptions (Graneheim and Lundman 2004; Berg 2009).

The research team included E.W., who is an endodontist with previous experience collecting qualitative data, resulting in several scientific publications. She is involved in undergraduate and postgraduate education on the topics of men’s violence against women and violence in close relationships, with associated external engagements. G.P. is a psychologist with extensive research experience in CSA and some experience in research based on qualitative data. S.M. and L.W. are both doctors of dental surgery, with limited research experience.

### Recruitment of Informants

Inclusion criteria for participation in an interview were 1) experience of sexual abuse, 2) awareness that this experience has manifested itself during dental visits, 3) ability to express themselves in Swedish, and 4) age ≥18 y. Selected for analysis in this study were informants with an experience of CSA.

**Figure 1.**
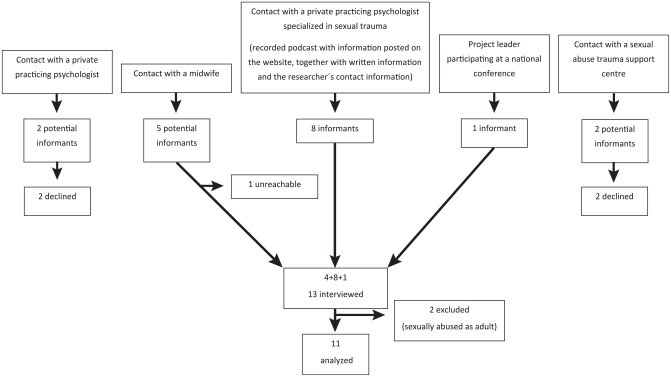
Flowchart illustrating the recruitment of informants to the interview.

Eighteen potential informants were contacted and purposively selected and recruited through various organizations and locations (Fig. 1). A total of 11 participated in the study (10 women, 1 man; age, 19 to 56 y). They lived in metropolitan areas, minor cities, as well as rural areas and had diverse social backgrounds: student, early retirement, working; single, partner/married; children, no children. Some indicated experiencing anorexia, bulimia, self-injury, drug abuse, prostitution, and imprisonment. A variety of sexual abuse was represented: sexualizing looks and allegations, isolated as well as repeated episodes of abuse over several years, with or without penetration. One or several male perpetrators were reported, mostly acquainted with the victims but sometimes strangers. All informants self-reported some degree of dental fear.

A personal meeting was arranged with each participant, during which written and oral information was provided and the topics to be covered during the interview were introduced. This initial meeting gave the 2 parties an opportunity to become acquainted and the informant a chance to ask questions about the project. The informants provided information about their psychotherapist, who was contacted by E.W. before the interview. They were given the opportunity of 2 sessions with their therapist at the expense of the research grant. This offer was accepted by 4 participants, while another 5 had already booked sessions with their psychotherapist.

All informants provided written informed consent for participation, and a semistructured in-depth interview was conducted once between April 10, 2017, and May 24, 2018, from 41 to 93 min in Swedish by E.W. at a location mutually agreed with the interviewee. There were 3 main topics:

1. “Please describe, in as much detail as possible, one or more dental appointments at which you were reminded of sexual abuse you have experienced.”
2. “How do you perceive the effect of sexual abuse on your oral health?”
3. “How do you perceive the effect of sexual abuse on your general health and quality of life?”

The interview topics were followed up with questions aimed at enhancing reflection and development of the narrative, allowing the informant’s story to be told. The interviews were recorded digitally and transcribed verbatim by an authorized secretary under a confidentiality agreement. All transcripts were listened to, reviewed for accuracy, and deidentified by E.W.

**Figure 2.**
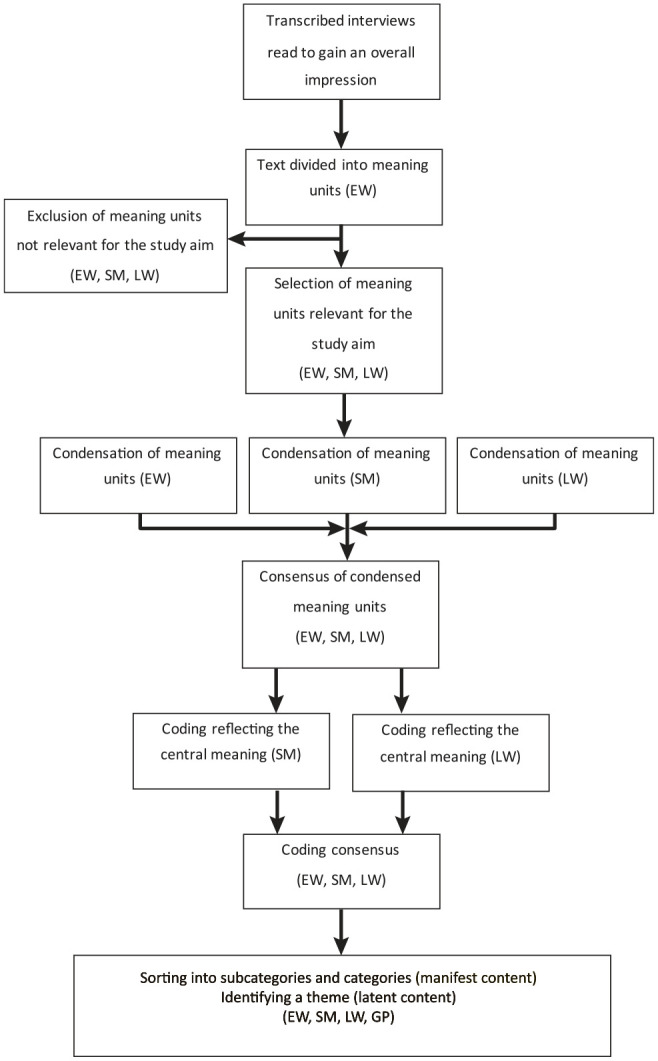
Flowchart illustrating the analysis process of the collected interview material.

As the analysis unit for this study included how the informants perceived their oral health (interview topic 2), the meaning units relevant to the study aim were identified and selected. The results of interview topic 1 have been reported (Wolf et al. 2020). The qualitative content was analyzed according to Graneheim and Lundman (2004; Table 1, Fig. 2). The codes were sorted into subcategories and categories representing the manifest content. An overall theme was identified, that is, an interpretation of the underlying message representing the latent content. The informants did not comment on the transcriptions or provide feedback on the findings. The Standards for Reporting Qualitative Research (SRQR) checklist was completed.

**Table 1.** Qualitative Content Analysis Process Used to Analyze Interviews and Extract Results.

Meaning Unit	Condensed Meaning Unit	Code	Subcategory	Category
I’m not going to have a single tooth left in my mouth soon. . . . I won’t. There are already 2 which are . . . broken, which are complete wrecks. . . . I just hate myself because it is the way it is . . . but . . . I don’t know what I’ll do.	I am not going to have a tooth left soon. I just hate myself but don’t know what I’ll do.	Self-punishment	Emotional barriers	Emotional significance
Going to the dentist was alright until I was . . . 8. . . . Because then I was the victim of abuse by yet another man. And there . . . it was very . . . yes, and a lot of it was oral. And so after that . . . I know that I had no . . . yes, going to the doctor’s went quite well like, or to the dentist in . . . until the age of 8. Because then . . . yes . . . because then it happened in his . . . garage and his basement and it was almost entirely oral abuse. So after that I didn’t allow anyone to come . . . E.W.: No one was allowed to come near you? No.	Going to the dentist was alright until I was 8. Then I was the victim of abuse by another man and a lot of it was oral. So after that I didn’t allow anyone to come (near).	Resistance to proximity	Dental appointments with complications	Obstacles to oral health

Two meaning units are condensed into more succinct formulations—specifically, condensed meaning units are presented with the corresponding code, subcategory, and category representing an example of the manifest content.

### Ethical Considerations

The study was conducted in accordance with the 1964 Declaration of Helsinki II (version 2002 revision; World Medical Association) and approved by the Ethics Review Board at Lund University (Dnr 2014/780). To help the informants deal with relived memories and emotions, professional support was available after the interview, if needed.

## Results

**Figure 3.**
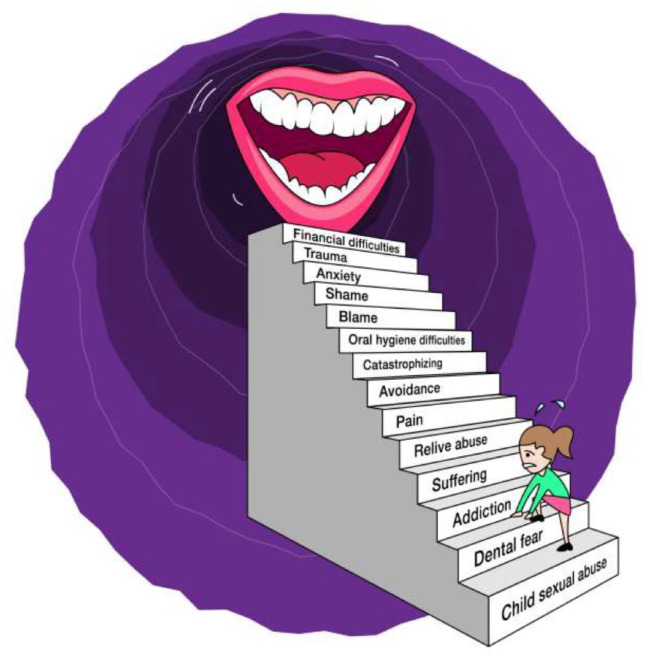
The obstacles representing the challenged conditions to maintain oral health after a sexual abuse experience.

The identified overall theme was “challenging conditions for maintaining oral health” (Table 2, Fig. 3), illustrating that the informants generally lived under circumstances that made it difficult to maintain adequate oral health. They stated that their mouth was a private and sensitive area and they wanted to retain strict control over whom they allowed to get close to this area. Oral health was considered important and closely associated with strong emotions. Though not considered by all informants to be directly related, the abuse experience was expressed as having had a direct or indirect impact on oral health.

The dental fear and the problems with my teeth . . . all of it can in fact really be traced back to . . . to my stepfather. Ehh . . . yes. (informant 10)

**Table 2.** Latent and Manifest Content.

Challenging Conditions for Maintaining Oral Health^a^	
Emotional Significance^b^	Obstacles to Oral Health^b^
Emotional barriers	Daily self-care with complications
Powerful relief	Dental appointments with difficulties

a The identified pattern, as described in a theme, covering the latent content.

b The category and subcategories covering the manifest content.

The first main category covering the manifest content was “the emotional significance,” with 2 subcategories: emotional barriers and powerful relief. The second main category was “the obstacles to oral health” with 2 subcategories: daily self-care with complications and dental appointments with difficulties (Table 2).

The main category “the emotional significance” covers a pattern of strong feelings about oral health and dental appointments. Negative feelings generally permeated the experiences, but there were also positive expressions, when a goal considered to be unachievable was in fact achieved.

The first subcategory, “emotional barriers,” comprised the informants’ expressions of shame, permeating their perception of their oral status. Informants stated that their oral status limited their enjoyment of everyday life and that they had often developed ways in which they could hide their teeth when they smiled and laughed. They were exhausted and ashamed of their dental status.

I’m not going to have a single tooth left in my mouth soon . . . I won’t. There are already 2 which are . . . broken, which are complete wrecks. . . . I just hate myself because it is the way it is . . . but . . . I don’t know what I’ll do. (informant 12)

A visit to the dentist could cause high levels of anxiety, including notions of the worst possible scenario and thoughts of catastrophe.

But the earth shook. (informant 13)

Mostly anxiety was induced when the participants postponed dental appointments or when they considered it impossible to cope with an appointment. They reported becoming physically ill prior to scheduled appointments, resulting in abandonment of treatment. In some cases anxiety about their dental status motivated them to keep the dental appointment, but they perceived the dental examination as judgmental and likened it to the abuse situation.

Because they can in fact make little comments about: “Yes, here you’ve been a bit careless.” So I am in fact judged. And that is something that has happened in abuse . . . when I was being abused. (informant 11)

Emotional barriers could be expressed as avoidance behavior: one did not want to be reminded of past abuse and so abstained from dental care.

That’s probably what frightens me most, that I might get stirred up again. Because I feel so well these days, you see. So I don’t want to . . . go back to how I used to be . . . so that I am reminded of . . . of this, you see. So that . . . that is probably the only danger I feel today, it is in fact . . . I feel too well to go to the dentist. That sounds awful. My mouth isn’t in good shape, but otherwise I have the best life I can imagine, helping other people, you see. (informant 9)

There were feelings of injustice about dental health: despite good self-care, there was still suffering and pain.

The subcategory “powerful relief” comprised expressions of relief after various forms of dental treatment had been achieved, as well as feelings of strength and pride when after several years the informant managed to seek dental care, handle the fear, and cope with treatment.

Yes, but the pride I felt in having done it, I managed to get through it and it didn’t kill me [*a few tears*]. (informant 13)

Informants with a history of dental fear, drug abuse, and major need for dental treatment could express great joy and gratitude toward the dentist after undergoing and coping with the treatment.

So she helped me. In fact she is the person who has helped me most in my life, a dentist. (informant 4)

Severe dental fear was expressed but followed by a great personal victory of having managed to endure the treatment.

The main category “the obstacles to oral health” highlighted the importance that the informants placed on oral health and the difficulties associated with achieving this. Although efforts were made to maintain oral health, even by former addicts during drug addiction periods, the participants expressed a desire for better oral hygiene, partly to maintain oral health but also as a means of avoiding the need for emergency dental care. The inconvenience of unhealthy teeth was additionally mentioned.

In the subcategory “daily self-care with complications,” toothbrushing was a major issue affecting self-care, and several months could pass without toothbrushing.

Toothbrushing, to like have anything in my mouth can be very sensitive. (informant 11)

Informants stated that difficulties associated with self-care were exacerbated by initiation of psychological treatment, when disclosure of CSA caused traumatic memories to resurface.

It was *really* hard. In fact I retched to the point where I almost vomited in fact. . . . In fact I had probably done that now and again beforehand too, but now it was every time. *Every* time I brushed my teeth, morning and evening. (informant 7)

The informants were self-critical and expressed that their self-care was inadequate for their perceived needs. They expressed that to some extent self-control facilitated self-care such as toothbrushing. Extensive drug abuse, reported by some, was described to have contributed to impaired oral hygiene and dental status.

I have in fact always been so very, very careful about my teeth . . . despite everything, eh. When I’ve been on narcotics, with narcotics. So even when I’ve been on narcotics and so on, I’ve always looked after my teeth. (informant 5)

The informants stated that periods of low self-esteem affected self-care, which was then not prioritized.

The second subcategory was “dental appointments with complications.” All informants self-reported some level of dental fear, mostly severe. At the same time, they noted a great need for dental treatment. Some were so limited by fear that it was unthinkable to seek dental care.

When I was last there . . . it is in fact . . . 7 or 8 months since I was sent a recall letter, and then I had broken off a piece of the tooth, which they had to repair. But then in fact they had to . . . open it up a little, or . . . yes, I don’t know how it works. [*Little laugh*] But it loosened 4 or 5 days later, and I haven’t been there . . . so now . . . now I have a very big hole. And I cannot . . . I cannot ring them up. I just can’t. (informant 11)

Difficulty in tolerating objects in the mouth and feelings of powerlessness in the dental setting led to the need to perform the dental treatment themselves. One had gone so far as to deal with the complaints herself.

But I also need to like replace the teeth that I’ve pulled out myself. . . . They are going to come out no matter how much it hurts. (informant 1)

Painkillers were used to alleviate toothache when professional dental care was unthinkable.

I have had lots of inflammation in the broken teeth. . . . If I take painkillers for a week, or aspirin, it settles down and then goes away. And then you have to squeeze out the pus, and that leaves a really “nice” taste in your mouth. But I’d rather put up with that. (informant 9)

The consequences of lack of dental care, often combined with financial constraints, led to emergency treatment. Some observed that attending only for emergency treatment meant that they eventually required more comprehensive treatment than if they had been regular attenders.

So in fact I never had . . . the financial means even to think about going. (informant 7)

The informants had in some cases requested, or had been offered and accepted, general anaesthesia as a facilitating shortcut.

I couldn’t see any other way out . . . other than a general anaesthetic. Because there, one is certainly powerless, when you have a general anaesthetic. But then I knew that everything would be done at the same appointment and I wouldn’t have to be tortured any further and so on. (informant 3)

General anaesthesia was, however, not always a positive experience: all trust disappeared when the dentist undertook more extensive treatment than had been agreed.

That I left the hospital dental clinic, that was just because once he gave me a general anaesthetic . . . I had a general anaesthetic and then he took the chance to take out 7 of my teeth. (informant 4)

Nitrous oxide sedation was sometimes appreciated as a shortcut to treatment.

I found out that hospital dentists had laughing gas and that it was for other . . . that it was for patients who had problems. So I went there and then I had laughing gas. (informant 4)

Sometimes, though, this did not work. It could be ineffective in cases of previous drug addiction, or the informant avoided sedation because it was reminiscent of the loss of control and vulnerability associated with the episodes of sexual abuse.

And then it was like yet another assault. Then quite suddenly I had to simply put on something that was . . . a mask, some sort of a mask. And I was to smell this [*breathes in through her nose*] which came rushing out, the laughing gas. And then I started to feel so strange from it, so that . . . I lost control . . . of myself, and then there was even more of this: “No, I won’t have this. I won’t let anyone do anything to me that I don’t know about.” (informant 1)

Other shortcuts were sought (e.g., not contacting the dental clinic but instead leaving it to the clinic to make the appointments).

## Discussion

This in-depth study of perceptions of oral health held by adult survivors of CSA disclosed and clarified major barriers that they encounter in trying to achieve an acceptable standard of oral health. They cited numerous impediments to their efforts to maintain oral hygiene and to have regular dental care. They reflected on their oral health with intense emotions, mainly negative but occasionally positive.

To capture the informants’ inner and subjective perspectives, qualitative content analysis was chosen (Graneheim and Lundman 2004). Their varied experiences of sexual abuse, social background, and life experience were in favor of broad access to information. The limited number of participants might mean that saturation was not fully achieved, which does not necessarily invalidate the findings but does indicate that the topic has not been fully explored (Morse and Field 1995; Boddy 2016). Rather than presenting definitive answers, the results contribute to a broader understanding by the dental profession of the care that those exposed to CSA receive, fulfilling the intentions stated in Swedish legislation: “to satisfy the patient’s need for security in care and treatment” (“SFS 1985:125” 1985).

Oral health was stated to be inadequate and to have a negative impact on daily life, whether or not the abuse experience was regarded as a contributing factor. Although oral health was self-reported as inadequate and not based on professional assessment, the results are in accordance with other studies showing that survivors of CSA have poorer oral health than unaffected individuals (Kundu et al. 2014). Difficulty chewing, teeth missing due to extraction, active dental disease, and oral pain mean that the criteria for oral health, as stated by the World Dental Federation (FDI 2016), are not met (Glick et al. 2016).

Some informants related their inadequate oral health to CSA. That difficulties in maintaining or achieving oral health might be related to the experience of CSA is exemplified by the finding of explicit expression of a direct association. A further example of a relation between CSA and obstacles regarding oral health, was the experience expressed by the informants of difficulty in maintaining daily oral hygiene when the sexual trauma had been disclosed and was under treatment by a therapist. Although the abuse experience is most likely not the only reason for the perceived oral health problems, it is certainly an important risk factor. Moreover, the self-reported drug abuse related to the development of dental fear (Willumsen 2001; Leeners et al. 2007; Fredriksen et al. 2020) sometimes contributes to the deterioration in oral health.

Given the powerful emotions expressed by the informants, it might be concluded that they regarded their oral health as important, though in many cases this had not been subjectively achieved. Negative feelings acting as barriers predominated the emotions reflected in the study, where guilt and shame may be of particular importance. Shame, in its negative forms, becomes a direct attack on the “self” (Tomkins 1987). The continuum of shame ranges from a mild sense of embarrassment to extreme humiliation, with more severe psychological consequences set along the progression of the continuum and aggravated by repetition (Tomkins 1987). The feeling of shame is indicated to be very painful (Lewis 1987; Dawson 1996): the person experiences loss of self-esteem in one’s own eyes and those of others (Lewis 1987). CSA often causes shame (MacGinley et al. 2019), and this is the case with poor oral health, not only in a social context (Sanders et al. 2006) but also in the dental setting (Wolf et al. 2020). This was confirmed in our study. With respect to shame, the relevance and impact of the dental care providers’ attitude toward a patient with poor oral health, who repeatedly cancels or fails to attend scheduled appointments, warrant further exploration. Shame can trigger a negative spiral (Scheff 1990) and is activated through criticism, condemnation, and threats of being rejected (Lewis 1987), situations that might arise in a dental encounter when a patient presents with poor oral health and inadequate oral hygiene (Wolf et al. 2021).

The intensity of the negative and positive emotions that the informants expressed in the oral health context might be of particular relevance, not only in relation to oral health per se but also with reference to the thought of seeking dental care. This might be interpreted as a sign that oral health is of great importance to quality of life, as noted earlier (Bernabe and Marcenes 2010; Kaprio et al. 2012; Jansson et al. 2014) and therefore of utmost importance. Some suggested a proactive approach by the dental practice: contacting the patient individually to schedule appointments, which might mean a deviation from regular routines to facilitate the achievement of acceptable oral health for those showing obvious or subtle signs (Wolf et al. 2020) of dental fear, such as repeated cancelation of appointments.

According to the fear acquisition theory (Rachman 1977), fear can be acquired through 3 pathways: 1) conditioning, acquired in an acute situation or produced by experiences of subtraumatic or even nontraumatic situations; 2) indirect conditioning, acquired through vicarious exposures; and 3) transmission of verbal information and instruction. The severity of fear depends on the magnitude of the stimulus, the duration, and the number of repetitions between the association of the fear experience and the stimulus (Rachman 1977). Fear is an essential response for survival, but it can be maladaptive, as when it results in avoidance of dental care. All 3 pathways may have relevance for the development of dental fear in survivors of CSA, although indirect conditioning is the one most directly associated with the abuse experience.

The informants’ reluctance to visit the dentist confirmed earlier results (Hakeberg et al. 2000). This and the occasionally pronounced wish for shortcuts to dental treatment, such as a mental escape through general anaesthesia and nitrous oxide sedation, might be interpreted as a fight-flight or freeze reaction (van der Kolk 2015). This concept is considered a necessary part of human nature to survive threats (van der Kolk 2015). For survivors of CSA, situations that are not dangerous per se can be perceived as life threatening, resulting in a nonfunctional escape (Shapiro and Dominiak 1990; Romans et al. 1999; Johnson et al. 2003), as in the dental surgery setting (Wolf et al. 2020). For clinicians, escape behavior warrants further investigation to improve dental care. This situation might provide an opportunity for the aware dental care provider in an atmosphere of alliance (Kranstad et al. 2019; Wolf et al. 2021) to bring to light a possible abuse experience previously undisclosed (Krug et al. 2002; Överlien 2004; Samelius et al. 2007; Priebe and Svedin 2008). If necessary, the patient could then be guided toward psychological treatment.

In this context, it is important to highlight not only organizational but also government initiatives intended to achieve the legislated standards of dental care in Sweden (“SFS 1985:125” 1985). An important step in this direction was the recent introduction (2018, 2019) of the new mandatory examination objective “Show knowledge of men’s violence against women and violence in close relationships” in 8 Swedish higher education courses in health care, including undergraduate dental and dental hygienist courses (“SFS 2017:857” 2017). It is hoped that this will result in greater awareness among graduating dental care providers of 1) physical and psychological (general and dental) health impacts of CSA on survivors (Kirkengen 2001; Leeners et al. 2007) and 2) response to treatment of patients who have experienced CSA. The aim is to achieve patient-centered individualized dental care (Kranstad et al. 2019; Wolf et al. 2021), leading to regular dental attendance and improved oral health.

Impediments to daily oral hygiene and a desire for better oral health were evident. All informants reported some degree of dental fear. Sometimes, with financial constraints, this had discouraged them from seeking regular dental care. Oral health was associated with strong feelings, such as shame, blame, anxiety, and sometimes pride. The findings, with those of other recently published studies (Kranstad et al. 2019; Fredriksen et al. 2020; Wolf et al. 2021), provide insights that support the importance of the need for improved dental care and better understanding by dental professionals of the needs of survivors of CSA. With few exceptions, CSA is illegal worldwide and undeserved by the victims. A person exposed to abuse and injured by another human being has the right to recover and, under Western law, the right to restitution (Spindel et al. 2000; UNICEF 1990).

## Author Contributions

E. Wolf, contributed to conception, design, data acquisition, analysis, and interpretation, drafted and critically revised the manuscript; S. Månsson, L. Wallin, contributed to data analysis and interpretation, drafted the manuscript; G. Priebe, contributed to data analysis and interpretation, drafted and critically revised the manuscript. All authors gave final approval and agree to be accountable for all aspects of the work.
